# Carbohydrate intake attenuates post-exercise plasma levels of cytochrome P450-generated oxylipins

**DOI:** 10.1371/journal.pone.0213676

**Published:** 2019-03-18

**Authors:** David C. Nieman, Nicholas D. Gillitt, Guan-Yuan Chen, Qibin Zhang, Camila A. Sakaguchi, Ella H. Stephan

**Affiliations:** 1 Human Performance Laboratory, Appalachian State University, North Carolina Research Campus, Kannapolis, North Carolina, United States of America; 2 Dole Nutrition Research Laboratory, North Carolina Research Campus, Kannapolis, North Carolina, United States of America; 3 UNCG Center for Translational Biomedical Research, University of North Carolina at Greensboro, North Carolina Research Campus, Kannapolis, North Carolina, United States of America; 4 Physical Therapy Department, Federal University of São Carlos, São Carlos, SP, Brazil; 5 Department of Nutrition, UNC Gillings School of Global Public Health, University of North Carolina-Chapel Hill, NC, United States of America; Victoria University, AUSTRALIA

## Abstract

**Introduction:**

Oxylipins are bioactive oxidation products derived from n-6 and n-3 polyunsaturated fatty acids (PUFAs) in the linoleic acid and α-linolenic desaturation pathways.

**Purpose:**

This study determined if carbohydrate intake during prolonged and intensive cycling countered post-exercise increases in n-6 and n-3 PUFA-derived oxylipins.

**Methods:**

The research design utilized a randomized, crossover, counterbalanced approach with cyclists (N = 20, overnight fasted state, 7:00 am start) who engaged in four 75-km time trials while ingesting two types of bananas (Cavendish, Mini-yellow), a 6% sugar beverage, and water only. Carbohydrate intake was set at 0.2 g/kg every 15 minutes, and blood samples were collected pre-exercise and 0 h-, 0.75 h-,1.5 h-, 3 h-, 4.5 h-, 21 h-, 45 h-post-exercise. Oxylipins were measured with a targeted liquid chromatography-multiple reaction monitoring mass spectrometric method.

**Results:**

Significant time effects and substantial fold-increases (immediately post-exercise/pre-exercise) were measured for plasma levels of arachidonic acid (ARA), eicosapentaenoic acid (EPA), docosahexaenoic acid (DHA), and 43 of 45 oxylipins. Significant interaction effects (4 trials x 8 time points) were found for plasma ARA (P<0.001) and DHA (P<0.001), but not EPA (P = 0.255), with higher post-exercise values found in the water trial compared to the carbohydrate trials. Significant interaction effects were also measured for 12 of 45 oxylipins. The data supported a strong exercise-induced increase in plasma levels of these oxylipins during the water trial, with carbohydrate ingestion (both bananas types and the sugar beverage) attenuating oxylipin increases, especially those (9 of 12) generated from the cytochrome P-450 (CYP) enzyme system. These trials differences were especially apparent within the first three hours of recovery from the 75-km cycling bout.

**Conclusions:**

Prolonged and intensive exercise evoked a transient but robust increase in plasma levels of oxylipins, with a significant attenuation effect linked to acute carbohydrate ingestion for 28% of these, especially those generated through the CYP enzyme system.

**Trial registration:**

ClinicalTrials.gov, U.S. National Institutes of Health, NCT02994628

## Introduction

Oxylipins are bioactive oxidation products derived from n-6 and n-3 polyunsaturated fatty acids (PUFAs) in the linoleic acid and α-linolenic desaturation pathways [1–3]. Oxylipins are not stored due to their potency, and are instead synthesized de novo in a tightly regulated manner [4]. Membrane phospholipid PUFAs are first released by phospholipase A_2_ (PLA_2_) in response to cell activation from various stress-related stimuli including injury or inflammation. Cyclooxygenase (COX), lipoxygenase (LOX), and cytochrome P450 (CYP) enzyme systems metabolize the released PUFAs into numerous and diverse oxylipins that act as autocrine and paracrine lipid mediators by binding to cell surface G protein-coupled receptors or to multiple intracellular and nuclear receptors such as peroxisome proliferator-activated receptor-γ (PPAR- γ) [4].

Recent advances in mass spectrometry equipment and analytical capacities have increased awareness of the vital regulatory roles of oxylipins in numerous physiological processes including cardiac function, vascular tone, blood coagulation, innate immune function, and inflammation [1,2,5]. The influence of various exercise workloads and diet interventions, obesity, and various disease states on oxylipin generation is an emerging field of scientific endeavor [6–10]. There is a growing awareness that metabolic, lifestyle, environmental, and physiological stresses can turn oxylipins from beneficial signaling agents into mediators of immune dysfunction, chronic inflammation, and other unfavorable responses [1,11–13].

Exercise-induced inflammation, oxidative stress, and muscle tissue injury result in a robust immune response involving granulocytes, monocytes, macrophages, and lymphocytes [14]. Limited evidence indicates that oxylipins are involved to some degree in regulating the transient immune and physiological responses to acute exercise bouts [15–18]. Each of the 12 PUFAs in the linoleic and ALA pathways is mobilized strongly from adipose tissue stores during intensive and prolonged exercise [19–23]. At the same time, a large number of exercise-related oxylipins are produced, many of which are stable enough to be measured in plasma during several hours of recovery.

In the first paper from this study, we reported that carbohydrate intake from bananas and the sugar beverage compared to water was associated with higher post-exercise plasma glucose and fructose, and lower biomarkers for inflammation including leukocyte counts, plasma 9- and 13-hydroxy-octadecadienoic acid (9+13 HODES), and IL-6, IL-10, and IL-1ra [24]. Carbohydrate compared to water intake also resulted in an extensive reduction in post-exercise shifts in numerous lipid super-pathway metabolites including ketones, glycerol, long-chain fatty acids, and 9,10 dihydroxy-octadecenoic acid (9,10-DiHOME) [20,24]. Linoleic acid is a direct precursor to 9+13 HODEs and 9,10-DiHOME which are stable and abundant oxylipin products in human plasma. 9+13 HODEs have emerged as important indicators of oxidative stress and inflammation following stressful exercise and in a wide variety of pathological conditions [22–25].

We sought to determine if carbohydrate intake during exercise countered other PUFA-derived oxylipins, a likely finding given their important immune and inflammation regulatory roles. One potential mechanism for the influence of carbohydrate intake on lipid-related metabolites during exercise is the associated increase in insulin that inhibits tissue triacylglycerol lipase, hormone sensitive lipase, and PLA_2_, thus reducing triacylglycerol breakdown and the release of free fatty acids into circulation [26,27]. Prolonged exercise increases PLA_2_ activity in muscle tissue, and the potential countermeasure influence of acute carbohydrate ingestion through related increases in insulin and other factors should result in a reduced release of oxylipins and inflammatory lipid mediators.

## Materials and methods

The protocol for this trial and supporting Consolidated Standards of Reporting Trials (CONSORT) checklist are available as Protocol S1 and Checklist S1, and were also included in the first publication [24]. Participants signed informed consent and study procedures were approved (24 February 2016, with closure on 11 November 2016) by the Institutional Review Board at Appalachian State University. Data were collected at the Human Performance Laboratory at the North Carolina Research Campus in Kannapolis, NC. The study was first submitted to ClinicalTrials.gov on November 24, 2015, but due to a communication error between the IRB office and the primary investigator, the ClinicalTrials.gov submission was not corrected, approved, and posted until December 16, 2016. The authors confirm that all ongoing and related trials for this intervention are registered.

**Fig 1:**
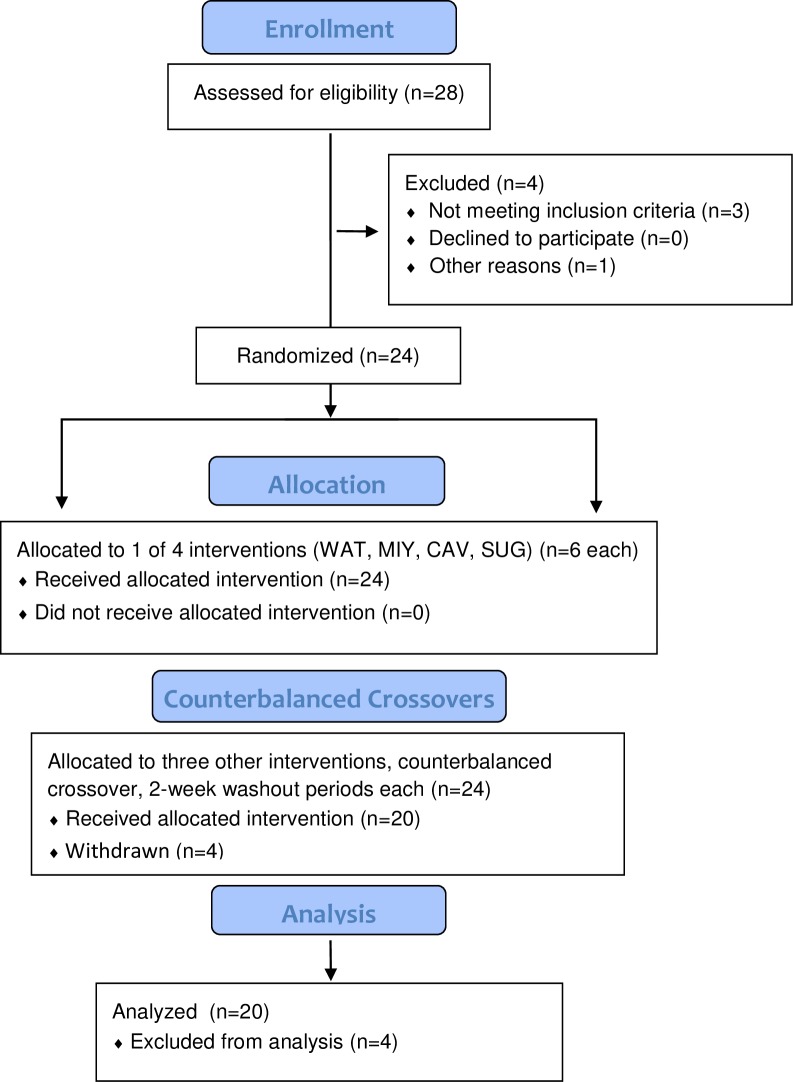
Study participant flow diagram. WAT = water trial; MIY = mini-yellow banana trial; CAV = Cavendish banana trial; SUG = sugar beverage trial. Four study participants randomized into the study failed to complete all four arms of the study (three due to changes in personal schedules and one to a training-related injury).

Information regarding study participants (N = 20 male and female cyclists, ages 22–50 years) and the research design were included in the first publication [24]. Briefly, this study utilized a randomized, crossover, counterbalanced approach with overnight fasted cyclists who engaged in the four 75-km time trials (2-week washout) while ingesting two types of bananas with similar carbohydrate content (Cavendish, mini-yellow), a 6% sugar beverage, and water only (Fig 1). Carbohydrate intake was set at 0.2 g/kg every 15 minutes. The volume of mini-yellow banana consumed was adjusted for the 5.4% higher sugar content, and the 6% sugar beverage was formulated with the same ratio of sucrose, fructose, and glucose (2:1:1) measured in freeze-dried Cavendish bananas pre-, mid- and post-study (Ultra-High Performance Liquid Chromatography, Refractive Index Detection, Agilent 1200 series, Santa Clara, CA). Blood samples were collected pre-exercise and 0 h-, 0.75 h-,1.5 h-, 3 h-, 4.5 h-, 21 h-, 45 h-post-exercise. The pre-exercise, 21 h- and 45 h-post-exercise samples were obtained from participants at ~7:00 am in an overnight fasted and rested state. No other restrictions on eating or exercise habits were applied during the two recovery days. During the 10-week period when data were being collected, participants maintained their typical training regimen, and avoided the use of vitamin and mineral supplements, herbs, and medications.

### Oxylipin analysis

Oxylipins were analyzed using a recently published liquid chromatography-multiple reaction monitoring-mass spectrometry (LC-MRM-MS) method which measures 131 endogenous oxylipins [28]. Briefly, 5 μL mixtures of deuterated oxylipin standards (Cayman Chemical, Ann Arbor, MI, USA) were spiked into 200 μL aliquot of human plasma collected from each study participant. The sample mixture was cleaned with 96-well HLB SPE cartridges (30 mg, Waters, Milford, MA) to remove proteins. The resultant eluents were dried and reconstituted in 50 μL of 50% methanol for the following LC-MRM-MS analysis.

LC-MRM-MS analysis was performed on a Vanquish UHPLC coupled with a Quantiva triple quadrupole mass spectrometer (Thermo Fisher Scientific, Haverhill, MA). A HSS T3 column (100 x 2.1 mm, 1.8 μm, Waters, Milford, MA) was employed for separation of analytes. MS parameter settings for MRM transitions and electrospray ionization as well as LC separation gradient settings were described in detail previously [28].

LC-MRM-MS datasets were processed with TraceFinder 4.1 (ThermoFisher Scientific), and the auto-integrated peaks were inspected manually. Concentrations of each oxylipin were determined from calibration curve of each analyte, which was constructed by normalizing to the selected deuterated internal standards followed by linear regression with 1/x weighting. A total of 45 oxylipins were quantified with less than 5% missing data, and 0.001 (mean LOQ for oxylipins with missing data) was substituted for missing values. Table 1 provides a list of the abbreviations and formal names for the 45 oxylipins, the fatty acid substrate, and the associated enzyme system. See also S1 Table.

**Table 1:** Abbreviations, formal names, substrate sources, and enzyme systems for the 45 oxylipins detected in this analysis.

Abbreviation	Formal name	Substrate	Enzyme system
5-iso PGF_2α_VI	(8ß)-5,9α,11α-trihydroxy-prostadienoic acid	ARA	Nonenzymatic
PGFM	13,14-dihydro-15-keto-prostaglandin F_2α_	ARA	COX/Nonenzymatic
TxB_2_	thromboxane B_2_	ARA	COX
tetranor-PGDM	9α-hydroxy-dioxodihydrotetranor-prostandioic acid	ARA	COX
12-HHTrE	12-hydroxy-heptadecatrienoic acid	ARA	COX
18-HEPE	18-hydroxy-eicosapentaenoic acid	EPA	COX
5-HETE	5-hydroxy-eicosatetraenoic acid	ARA	LOX
8-HETE	8-hydroxy-eicosatetraenoic acid	ARA	LOX
9-HETE	9-hydroxy-eicosatetraenoic acid	ARA	LOX
11-HETE	11-hydroxy-eicosatetraenoic acid	ARA	LOX
12-HETE	12-hydroxy-eicosatetraenoic acid	ARA	LOX
15-HETE	5-hydroxy-eicosatetraenoic acid	ARA	LOX
tetranor 12-HETE	8-hydroxy-hexadecatrienoic acid	ARA	LOX
5-oxo-ETE	5-oxo-eicosatetraenoic acid	ARA	LOX
9-oxo-ODE	9-oxo-octadecadienoic acid	Linoleic Acid	LOX
13-oxo-ODE	13-oxo-octadecadienoic acid	Linoleic Acid	LOX
9-HODE	9-hydroxy-octadecadienoic acid	Linoleic Acid	LOX
13-HODE	13-hydroxy-octadecadienoic acid	Linoleic Acid	LOX
5-HETrE	5-hydroxy-eicosatrienoic acid	Dihomo-γ-Linolenic Acid	LOX
8-HETrE	8-hydroxy-eicosatrienoic acid	Dihomo-γ-Linolenic Acid	LOX
15-HETrE	15-hydroxy-eicosatrienoic acid	Dihomo-γ-Linolenic Acid	LOX
9-HOTrE	9-hydroxy-octadecatrienoic acid	α-Linolenic Acid	LOX
13-HOTrE	13-hydroxy-octadecatrienoic acid	α-Linolenic Acid	LOX
5-HEPE	5-hydroxy-eicosapentaenoic acid	EPA	LOX
12-HEPE	12-hydroxy-eicosapentaenoic acid	EPA	LOX
15-HEPE	15-hydroxy-eicosapentaenoic acid	EPA	LOX
4-HDoHE	4-hydroxy-docosahexaenoic acid	DHA	LOX
8-HDoHE	8-hydroxy-docosahexaenoic acid	DHA	LOX
10-HDoHE	10-hydroxy-docosahexaenoic acid	DHA	LOX
13-HDoHE	13-hydroxy-docosahexaenoic acid	DHA	LOX
14-HDoHE	14-hydroxy-docosahexaenoic acid	DHA	LOX
16-HDoHE	16-hydroxy-docosahexaenoic acid	DHA	LOX
17-HETE	17-hydroxy-eicosatetraenoic acid	ARA	CYP
20-HETE	20-hydroxy-eicosatetraenoic acid	ARA	CYP
8,9-DiHETrE	8,9-dihydroxy-eicosatrienoic acid	ARA	CYP
11,12-DiHETrE	11,12-dihydroxy-eicosatrienoic acid	ARA	CYP
14,15-DiHETrE	14,15-dihydroxy-eicosatrienoic acid	ARA	CYP
20-COOH-AA	20-carboxy arachidonic acid	ARA	CYP
18-HETE	18-hydroxy-eicosatetraenoic acid	ARA	CYP
19-HETE	19-hydroxy-eicosatetraenoic acid	ARA	CYP
9,10-DiHOME	9,10-dihydroxy-octadecenoic acid	Linoleic Acid	CYP
12,13-DiHOME	12,13-dihydroxy-octadecenoic acid	Linoleic Acid	CYP
9,10-EpOME	9,10-epoxy-octadecenoic acid	Linoleic Acid	CYP
20-HDoHE	20-hydroxy-docosahexaenoic acid	DHA	CYP
19,20-DiHDPA	19,20-dihydroxy-docosapentaenoic acid	DHA	CYP

### Statistical analysis

Data are presented as mean±standard error (SE). The study participant number (N = 20) provided 84% power to detect a difference with an effect size 0.7 at alpha 0.05 using two sided paired t-tests. Data were checked for normality of the residuals using Q-Q plots. Oxylipin data were analyzed using the generalized linear model (GLM), and a 4 (condition) x 8 (time) repeated-measures ANOVA, within-participants design (IBM SPSS Statistics for Windows, Version 24.0, IBM Corp, Armonk, NY, USA). Changes over time within conditions were contrasted between trials using paired t-tests, with the alpha level set at P≤0.0125. Principle component analysis (PCA) and heatmap analysis of the data were performed with the R programming language (https://www.r-project.org/, version 3.3.1). The pheatmap package (1.0.8) was used to generate the heat map using Z scores (https://cran.r-project.org/web/packages/pheatmap/).

## Results

The analysis included 20 male cyclists (14 males, 6 females) who successfully adhered to all aspects of the study design. Study participant characteristics and performance data for the four 75-km cycling bouts are described in the first paper published in this journal [24]. Briefly, the average age of the study participants was 39.1±2.4 years, with a VO_2max_ of 46.9±1.9 ml^.^kg^.-1^min^-1^ and body fat of 19.3±1.5%, with no significant differences between male and female cyclists. Performance times (180±4.4 minutes, average of the four trials), absolute oxygen consumption (2.50±0.9 L/min), heart rates (141±3.0 beats/minute), the rating of perceived exertion (RPE) (13.6±0.3), and plasma volume shift (-11.1±1.3%) did not differ during the two banana and sugar beverage trials compared to the water condition.

**Fig 2:**
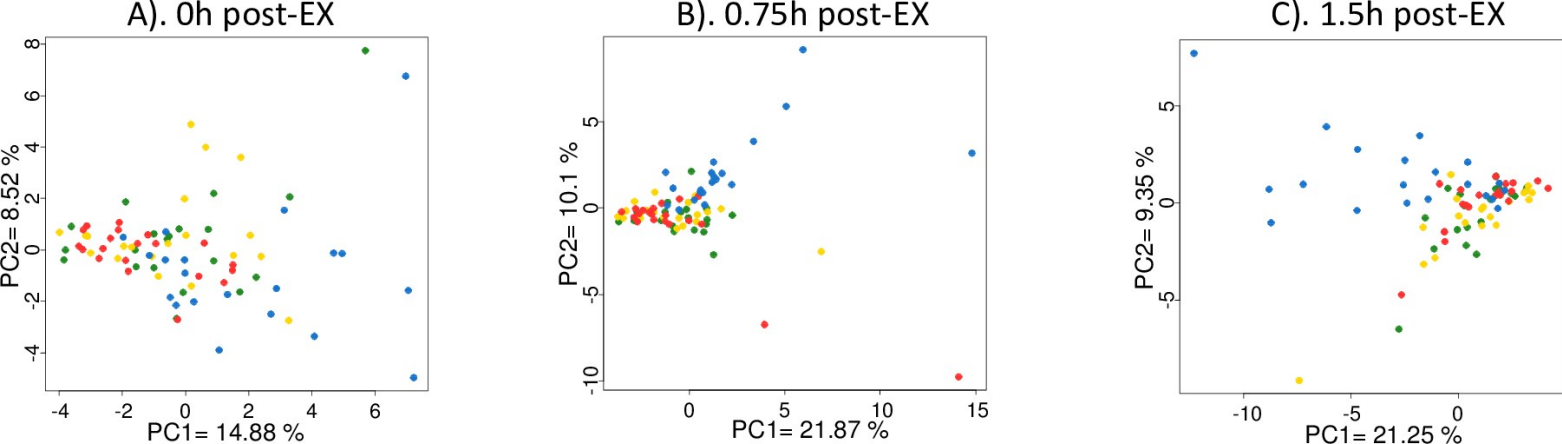
PCA analysis of data during the first 1.5 h recovery from 75-km cycling. A). Immediately post-exercise; B). 0.75 h post-exercise; C). 1.5 h post-exercise. The data support a distinct difference between the water trial and the three carbohydrate-based trials. Blue = water trial; Red = sugar beverage trial; Green = Cavendish banana trial; Yellow = Mini-yellow banana trial.

Fig 2 depicts the PCA analysis for samples across the four trials during the first 1.5 h recovery. The data indicate a distinct difference between the water trial and the three carbohydrate trials (two banana trials and the sugar beverage trial).

**Fig 3:**
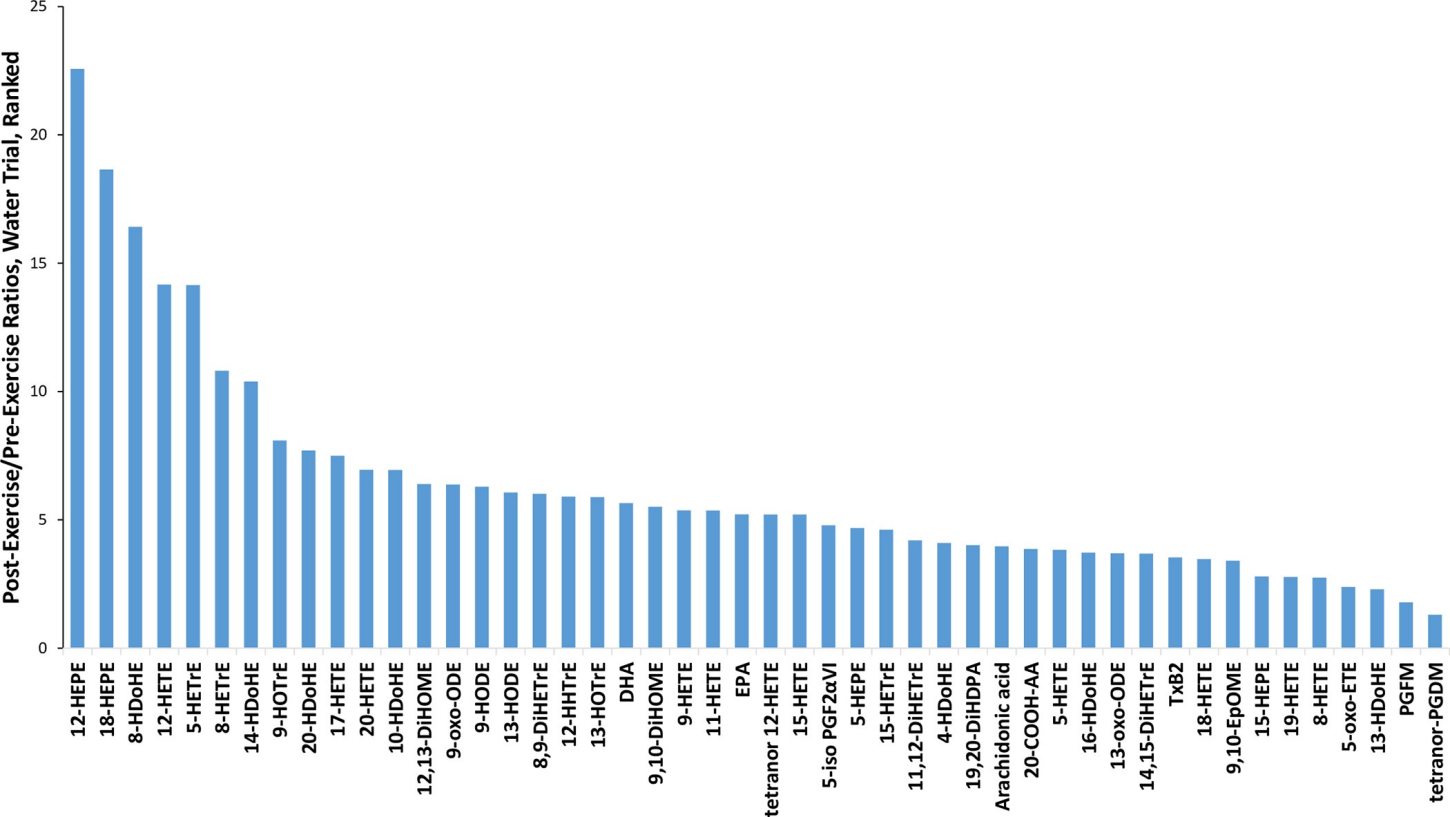
Ratio values (immediate post-exercise/pre-exercise) for N = 45 oxylipins, ARA, EPA, and DHA using data from the water trial. Significant time effects (P<0.05) were measured for each except 5-oxoETE (P = 0.139) and tetranor PGDM (P = 0.267).

Fig 3 depicts the fold-increases (immediately post-exercise/pre-exercise) for plasma levels of ARA, EPA, DHA, and 45 oxylipins. Significant time effects (P<0.05) were measured for each except 5-oxo-ETE (P = 0.139) and tetranor PGDM (P = 0.267).

**Fig 4:**
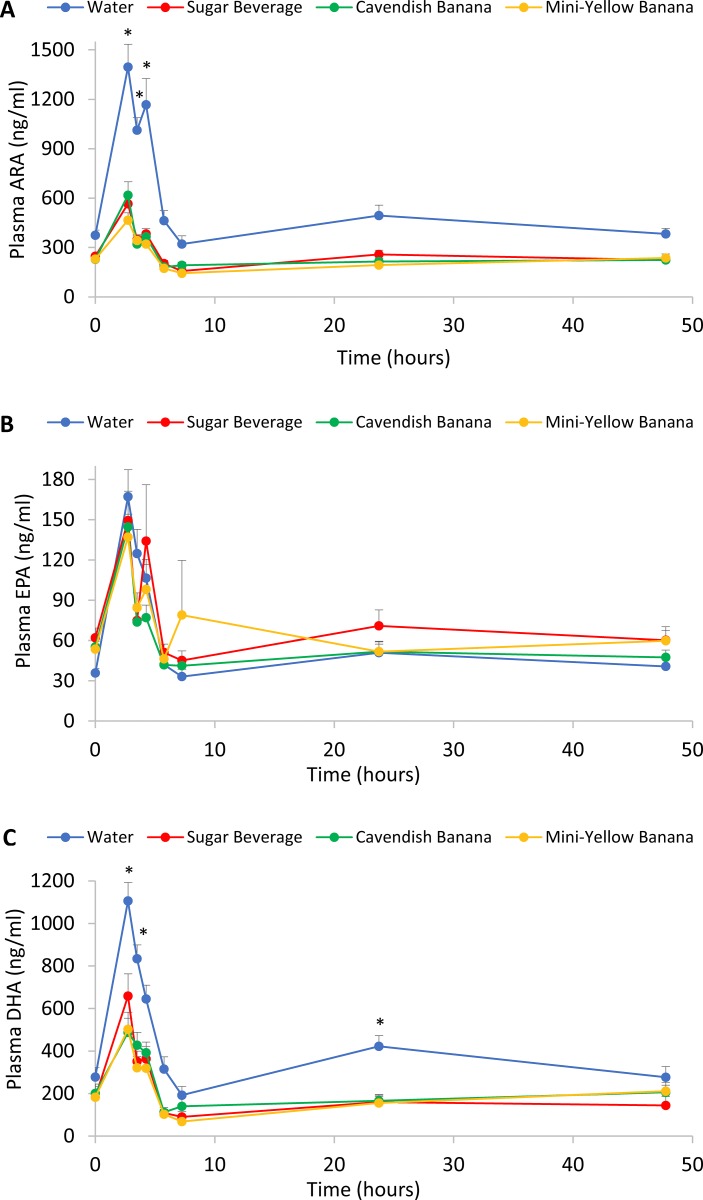
A) Arachidonic acid (ARA; 20:4n-6), interaction effect, P<0.001; B) Eicosapentaenoic acid (EPA; 20:5n-3), interaction effect, P = 0.255; C) docosahexaenoic acid (DHA; 22:6n-3), interaction effect, P<0.001. *P<0.0125, change from pre-exercise in the water trial compared to the carbohydrate trials (sugar beverage, Cavendish and Mini-yellow bananas combined). The X axis is on a continuous time scale, with blood sampling time points noted by the markers on the line graphs. Lunch was served after the 1.5 h blood sample (i.e., at 4.25 h on the time scale).

Fig 4 depicts trial differences over time for three of the oxylipin substrate lipids: ARA, EPA, and DHA. Significant interaction effects were found for plasma ARA (P<0.001) and DHA (P<0.001), but not EPA (P = 0.255), with higher post-exercise values found in the water trial compared to the carbohydrate trials.

**Fig 5:**
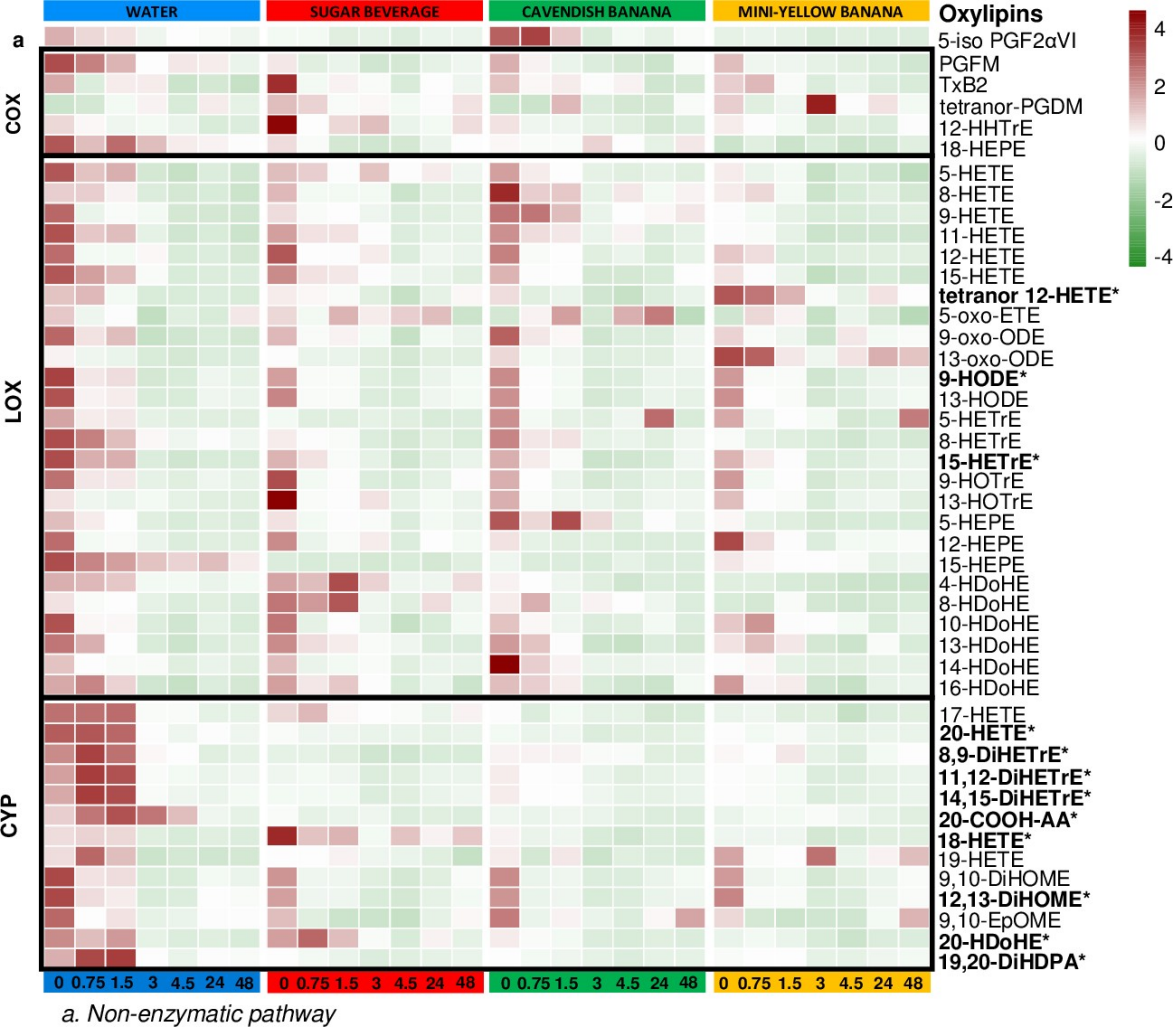
Heat map displaying oxylipin responses to exercise (ratios using pre-exercise values) in each of the four trials using Z scores. The data support a strong exercise-induced increase in plasma oxylipins during the water trial, with carbohydrate ingestion attenuating oxylipin increases, especially those generated from the P-450 cytochrome enzyme system. * P<0.05, interaction effect.

The heat map (Fig 5) displays plasma oxylipin responses to exercise (ratios using pre-exercise values) in each of the four trials. Z scores were used to display the data across rows. Significant interaction effects using repeated measures ANOVA were measured for 12 of 45 oxylipins, and the data support a strong exercise-induced increase in plasma levels of these oxylipins during the water trial, with carbohydrate ingestion (both bananas and the sugar beverage) attenuating oxylipin increases, especially those (9 of 12) generated from the CYP enzyme system. Of the 9 CYP-generated oxylipins attenuated post-exercise during the carbohydrate trials, six were from ARA (18-HETE, 20-HETE, 20-COOH-AA, 8,9-DiHETrE, 11,12-DiHETrE, 14,15-DiHETrE), two from DHA (20-HDoHE, 19,20-DiHDPA), and one from linoleic acid (12,13-DiHOME).

**Fig 6:**
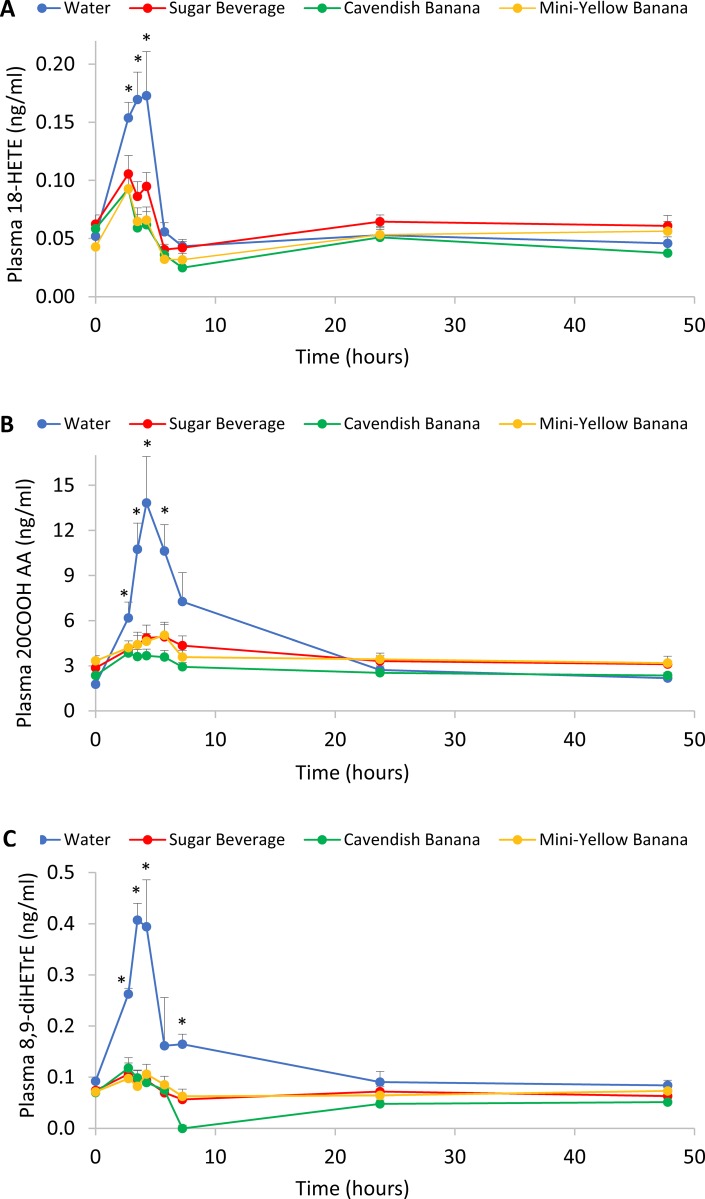
A) 18-HETE; B) 20-COOH-AA; C) 8,9-DiHETrE. *P<0.0125, change from pre-exercise in the water trial compared to the carbohydrate trials (sugar beverage, Cavendish and mini-yellow bananas combined). The X axis is on a continuous time scale, with blood sampling time points noted by the markers on the line graphs. Lunch was served after the 1.5 h blood sample (i.e., at 4.25 h on the time scale).

**Fig 7:**
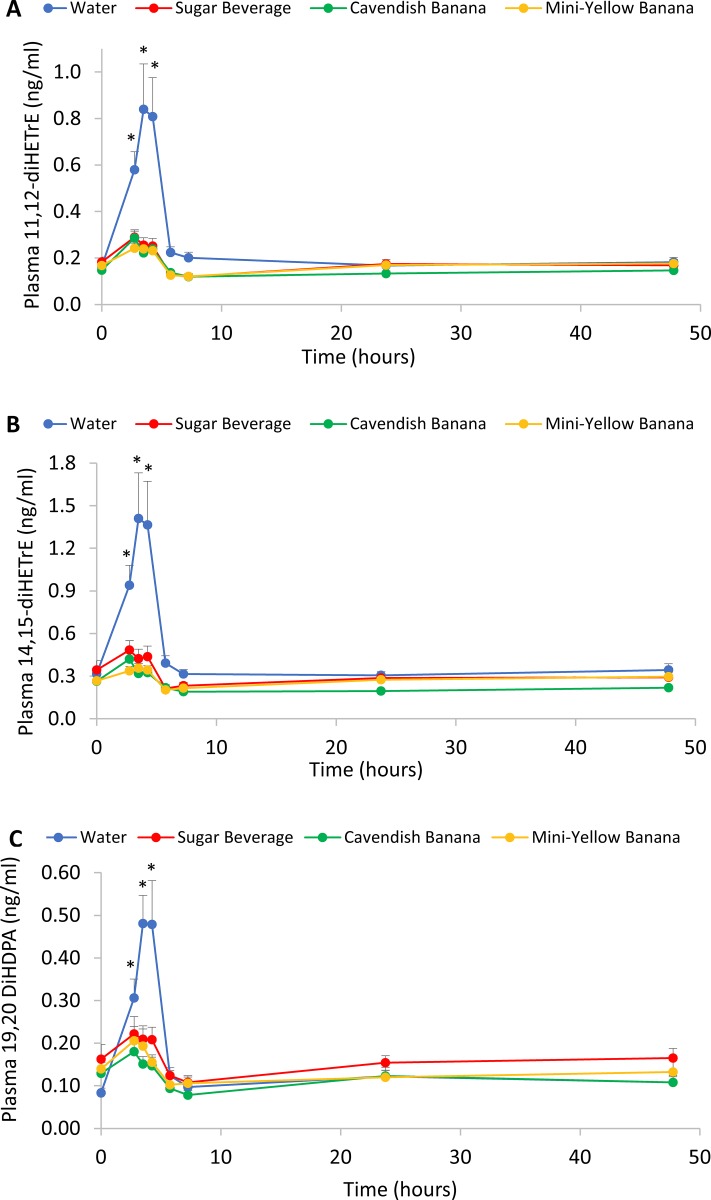
A) 11,12-DiHETrE; B) 14,15-DiHETrE; C) 19,20 DiHDPA. *P<0.0125, change from pre-exercise in the water trial compared to the carbohydrate trials (sugar beverage, Cavendish and mini-yellow bananas combined). The X axis is on a continuous time scale, with blood sampling time points noted by the markers on the line graphs. Lunch was served after the 1.5 h blood sample (i.e., at 4.25 h on the time scale).

Figs 6 and 7 depict trial differences over time for 6 of the 9 CYP-generated oxylipins. These data support strong trial differences between the water and carbohydrate trials, especially within the first three hours post-exercise.

## Discussion

As reported previously [24], regardless of the carbohydrate source (bananas or the 6% sugar beverage), carbohydrate intake at a rate of 0.2 g/kg every 15 minutes during 75 km cycling was associated with higher post-exercise plasma glucose and fructose, reduced plasma cortisol levels, diminished perturbation in lipid-related metabolites, and lower inflammation as assessed by total leukocyte and neutrophil counts, 9+13 HODEs, and plasma IL-6, IL-8, IL-10, and IL-1ra. In this analysis, we hypothesized that carbohydrate intake during exercise would attenuate post-exercise increases in plasma levels of n-6 and n-3 PUFA oxylipins. The data supported large post-exercise increases in plasma concentrations for 43 of 45 oxylipins, with a substantial attenuation linked to carbohydrate intake (bananas and sugar beverage) for 28% of these, especially those generated from the CYP enzyme system. The carbohydrate influence was evident during the first three hours of recovery from the 75-km cycling bout.

The oxylipin response to exercise is a new area of scientific endeavor, and the data from this study indicate that prolonged and intensive aerobic exercise causes large increases in a diverse range of plasma oxylipins generated from linoleic acid, dihomo-γ-linolenic, ARA, α-linolenic, EPA, and DHA substrates through COX, LOX, and CYP enzyme systems. Post-exercise plasma concentrations were highest for oxylipins generated from linoleic acid including 9,10-EpOME, 13-HODE, 9-HODE, 12,13-DiHOME, 9,10-DiHOME, 9-oxo-ODE, and 13-oxo-ODE, ARA (12-HETE, 20-COOH-AA, and 15-HETE), DHA (14-HDoHE), EPA (12-HEPE), and α-linolenic acid (13-HOTrE). 9+13-HODEs are stable oxidized metabolites that are secreted by a variety of cells and have been linked to multiple pathological conditions [22,23,25]. 9+13-HODEs function as biomarkers for both oxidative stress and inflammation, and respond to lifestyle interventions such as weight loss. 9,10- and 12,13-DiHOMEs are PPAR ligands with wide-ranging effects including stimulation of neutrophil chemotactic activity [29,30]. The HODEs and DiHOMEs can be detected using global metabolomics because of their high plasma concentrations, with targeted lipidomics required for most of the other oxylipins measured in this study.

In accordance with other studies, the largest number of oxylipins following 75-km cycling came from the n-6 PUFA substrate ARA, and these eicosanoids included at least 15 varieties of HETEs and DiHETrEs from LOX and CYP enzymes [15–17,31]. Although more exercise-based research is needed, the HETEs and DiHETrEs may have multiple potential roles during exercise recovery including the regulation of leukocyte migration and chemotaxis, macrophage efferocytosis and tissue repair, inflammation, PPAR activation, vascular tone, and platelet regulation [1,32]. The post-exercise fold increases in plasma oxylipins measured in this study far exceeded what has been reported following leg resistance exercise (3 leg exercises, 3 sets, 8–10 repetitions) [16].

The LC-MS/MS measurement system and assay utilized in this study can measure plasma oxylipin concentrations down to the 1 pg/ml level [28]. Despite having standards for most of the important specialized pro-resolving mediators (SPMs), none were detected in pre- and post-exercise samples except for a small number with maresin-1 (post-exercise). Although omega-3-derived SPMs have a well-defined role in inflammation resolution, these oxylipins may not accumulate in the plasma or tissue during and following exercise. SPMs are involved in the movement and function of neutrophils and macrophages [5]. Neutrophils accumulate in muscle tissue soon after intensive exercise, rapidly undergo apoptosis, and are cleared by macrophages [14,33]. This process involves the simultaneous integration of many different signaling processes, of which SPMs are just one. Our data indicate that SPMs do not accumulate in plasma even after prolonged and intensive cycling, and more research is needed at the muscle tissue level to determine if SPMs play a role in inflammation resolution in endurance-trained study participants.

COX enzymes generate a variety of prostaglandins and thromboxanes from ARA, EPA, adrenic acid, and dihomo-γ-linolenic acid including 1-, 2-, 3- and dihomo-2-series prostanoids [2]. ARA is the substrate utilized by the COX pathway to generate PGG_2_ which is quickly converted to PGH_2_, and then other oxylipins including PGD_2_, PGE_2_, PGI_2_, PGA_2_, PGF_2α_, PGB_2_, PGI_2_, TxA_2_, TxB_2_, and multiple downstream metabolites. Despite the importance of prostaglandins and thromboxanes as signaling molecules in muscle during recovery from intensive exercise [17,18], and the accurate and reliable LC-MRM-MS system used in this study, only four COX-ARA metabolites were detectable in plasma including TxB_2_ (the inactive metabolite of TxA_2_), PGFM (rapidly metabolized from PGF_2α_), tetranor PGDM (major metabolite of PGD_2_), and 12-HHTrE (produced from PGH_2_ with TxA_2_). A variety of prostaglandins have been measured in post-exercise muscle biopsy samples, with some reporting elevations in plasma for PGE_2_ and other prostaglandins using ELISA-based methods [17]. LC-MRM-MS is the best current methodology for measuring COX-ARA metabolites [28], and our data indicate that little accumulation of these metabolites occurred in human plasma following the cycling bout. We previously reported that banana ingestion during 75-km cycling increased post-exercise plasma levels of banana metabolites that were linked to a reduction in COX-2 mRNA expression in cultured THP-1 monocytes [24]. We expected to see reductions in plasma COX-ARA metabolites in the banana versus water and carbohydrate trials, but the data did not support this hypothesis, in part due to the limited presence of these metabolites in plasma following vigorous and prolonged cycling.

Carbohydrate intake strongly countered the mobilization of ARA and DHA, and the generation of oxylipins through the CYP enzyme system following the 75-km cycling bout. This is a novel finding that will require additional research to determine underlying mechanistic pathways. The findings imply that PLA_2_ and CYP activities were reduced with carbohydrate intake during and after exercise when blood sugars and insulin were higher, and inflammatory cytokines such as IL-6 and IL8 were lower, compared to the water trial. PLA_2_ is a group of enzymes that hydrolyze phospholipids to yield fatty acids and lysophospholipids, and are the initial, rate-limiting step of ARA metabolism leading to the production of bioactive lipid mediators [34]. Cytosolic PLA_2_ (cPLA_2_) exhibits preference for hydrolysis of ARA from phospholipid substrates, and expression is induced through a number of signaling pathways activated by acute exercise including MAPK/ERK (mitogen-activated protein kinases/ extracellular signal-regulated kinases), and transcriptional activators such as nuclear factor-kappa B (NF-κB) and proinflammatory cytokines [35,36]. Post-exercise plasma ARA levels were significantly reduced with carbohydrate intake, and these data imply that PLA_2_ activity was attenuated, perhaps in part due to higher glucose and insulin levels as extrapolated from diabetes-based investigations [26,37]. In a previous study conducted by our research group utilizing the same exercise model, we showed that serum insulin levels increased 29% after 75-km cycling with carbohydrate compared to a decrease of 28% with water only [20].

The epoxyeicosatrienoic acids (EETs) are formed by the metabolism of ARA by a specific subset of CYP enzymes called epoxygenases. The EETs are rapidly converted to dihydroxy-eicosatrienoic acids (DiHETrEs) by soluble epoxide hydrolase (sEH), and is consistent with our findings that showed no accumulation of EETs in the cyclists' post-exercise plasma [38]. The influence of exercise on CYP epoxygenases and sEH is currently unknown, but the data from this study suggest increased sEH activity with exercise that can be largely negated through carbohydrate intake. In comparison to the cardio-protective and anti-inflammatory actions of EETs, DiHETrEs exert proinflammatory effects and promote the chemotaxis response of human monocytes to MCP-1 [39]. Thus the sizeable countermeasure effect of carbohydrate intake on post-exercise plasma levels of DiHETrEs is consistent with other related anti-inflammatory responses (e.g, reductions in blood neutrophils, IL-6, and other cytokines). EETs play a role in reducing, resolving, and limiting inflammation, and EET activity may increase with carbohydrate intake during exercise as less is converted by sEH to DiHETrEs. The activation of EET activity through sEH inhibition is an active area of research due to other related benefits in glucose metabolism, insulin sensitivity, endothelial function, and lipid metabolism [10].

## Conclusions and application

Prolonged and intensive exercise evoked a transient but robust increase in plasma levels of numerous and diverse oxylipins, with a strong attenuation effect linked to acute carbohydrate ingestion for those generated through the CYP enzyme system. The data support both exercise and carbohydrate intake influences on PLA_2_, CYP epoxygenases, and sEH enzyme activities, but this must be verified in future investigations. There are multiple proposed roles for oxylipins during recovery from prolonged and intensive exercise, and the large effect of acute carbohydrate ingestion in lowering CYP-generated lipid mediators (including DiHETrEs) may promote EET anti-inflammatory responses and improved metabolic recovery. SPMs were not detected reliably in the plasma samples of the cyclists, calling into question their importance in resolving inflammation after heavy exertion.

## Supporting information

S1 Protocol Human research protection application and protocol summary. (PDF)

S1 Checklist CONSORT checklist. (DOC)

S1 Table Oxylipin data. (XLSX)
